# From Inhibition to Degradation: Can Galectin-3 Be Drugged by Heterobifunctional Degraders?

**DOI:** 10.3390/molecules31213853

**Published:** 2026-10-26

**Authors:** Edvin Purić

**Affiliations:** Department of Pharmaceutical Chemistry, Faculty of Pharmacy, University of Ljubljana, Aškerčeva Cesta 7, 1000 Ljubljana, Slovenia; edvin.puric@ffa.uni-lj.si

**Keywords:** cancer, fibrosis, galectin-3, lectins, PROTAC, targeted protein degradation

## Abstract

Several nanomolar galectin-3 inhibitors have advanced to clinical evaluation, which suggests that galectin-3 is a druggable target. These small-molecule inhibitors act by binding to the target protein and rely on an occupancy-driven mode of action that may not fully address intracellular functions of galectin-3. Proteolysis-targeting chimaeras (PROTACs) represent an alternative therapeutic modality, enabling intracellular target depletion with an event-driven mode of action. Galectin-3 is present both intracellularly and extracellularly; however, PROTACs can access only the intracellular pool, raising the question of whether selective intracellular degradation can provide sufficient therapeutic benefit. This perspective examines heterobifunctional galectin-3-targeting molecules composed of a glycomimetic ligand, an E3 ligase-recruiting ligand, and a linker. Their structures, degradation-related data, and mechanisms are critically assessed. Key challenges, including linker optimisation, ligand affinity, and intracellular target accessibility, are discussed. A very recent study demonstrated potent and selective galectin-3 degradation and expanded targeted protein degradation to structurally challenging lectins. Overall, galectin-3 represents a proof-of-principle that galectins undergoing intracellular–extracellular trafficking may potentially be degraded by PROTACs.

## 1. Introduction

Galectins are β-galactoside-binding lectins involved in cell–cell and cell–matrix interactions, glycoprotein trafficking, and immune signalling [1,2]. Among them, galectin-3 is a particularly compelling therapeutic target given its multifunctional roles in cancer, fibrosis, and inflammatory diseases [1,3,4,5,6]. It is overexpressed in many cancers including breast, colorectal, lung, pancreatic cancer and melanoma, while in fibrotic diseases such as idiopathic pulmonary fibrosis, it promotes fibroblast activation, proliferation, and extracellular matrix deposition [4,5,6]. Its secretion by macrophages and monocytes and localisation across intracellular and extracellular compartments underline its central role in linking immune dysregulation with tissue remodelling and chronic inflammation [7].

Most therapeutic efforts have focused on small-molecule inhibitors and glycomimetics designed to disrupt its carbohydrate-recognition domain binding to native ligands (CRD), yielding clinical candidates such as Selvigaltin (GB1211) and TD139/GB0139 [8,9,10]. Apart from small-molecule inhibitors, a polysaccharide polymer Belapectin (GR-MD-02) developed by Galectin Therapeutics is currently in Phase 2 clinical trial for the treatment of MASH cirrhosis [11]. However, these approaches remain limited by the occupancy-driven pharmacological mode of action. Moreover, galectin-3 engages in protein–protein interactions (PPIs) and intracellular signalling events that are unlikely to be fully blocked by competitive inhibitors alone, highlighting the need for alternative modalities capable of more robust and sustained target suppression.

Targeted protein degradation offers such an alternative by enabling the selective elimination of disease-relevant proteins rather than transient inhibition of their activity. Proteolysis-targeting chimaeras (PROTACs) are heterobifunctional molecules consisting of a ligand for the protein of interest (POI) connected via a linker to an E3 ubiquitin ligase-recruiting ligand [12,13]. PROTACs promote the POI and E3 ligase to form a ternary complex, which initiates the target protein ubiquitination and subsequent proteasomal degradation, resulting in an event-driven pharmacological mode of action. In general, PROTACs have higher molecular weight (typically between 700 and 1100 Da), more hydrogen bond-donors and acceptors, and a larger topological polar surface area (TPSA) compared with small-molecule inhibitors. Importantly, PROTACs require only transient binding to the target protein to initiate degradation, which may help overcome the relatively weak binding affinities of many galectin ligands—though the high polar surface area of galactose-based scaffold limits cell permeability and constrains linker design [14].

Strikingly, a patent by Liu et al. proposes extending the PROTAC strategy to galectin-3, despite the absence of corresponding peer-reviewed studies at the time. It discloses bifunctional compounds that combine a galectin-3-binding moiety with E3 ubiquitin ligase-binding ligands, consistent with canonical PROTAC design principles. These molecules are explicitly proposed as galectin-3 degraders or inhibitors, introducing a conceptual shift from target inhibition to target depletion. Despite the extensive chemical matter disclosed and the recognised relevance of galectin-3 in cancer, fibrosis, and inflammatory diseases, peer-reviewed evidence for galectin-3 degradation and mechanistic validation of galectin-targeting bifunctional molecules initially remained unavailable. This changed recently with the publication of a peer-reviewed study by Liu et al., which validated and extended the concept by identifying highly potent degraders and demonstrating their activity in cellular environments [7,15].

## 2. Chemical Design of Galectin-Targeting Bifunctional Degraders

**Figure 1:**
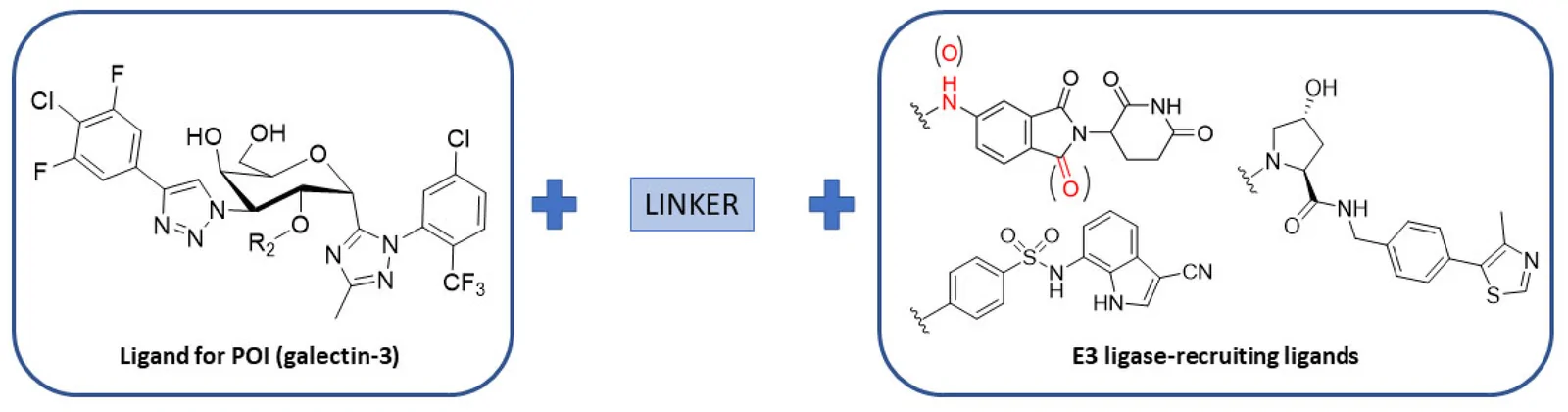
A general structure of galectin-3-targeting heterobifunctional degraders from the patent. A broad range of linkers were used in the synthesis of heterobifunctional molecules. The patent incorporates substituted galactopyranoside-like motifs functionalized with aromatic heterocycles, including triazole, phenyl rings, and halogenated substituents. All of them are known to engage subsites within the CRD and enhance binding affinities through hydrophobic contacts, halogen bonding, and π-π/cation-π interactions.

The patent by Liu et al. (WO2021/242982) discloses an extensive series of bifunctional molecules designed to target galectin-3 by combining a high-affinity galectin-binding ligand with an E3 ubiquitin ligase-recruiting ligand, connected via chemically diverse linkers (Figure 1) [7]. In contrast, the subsequent peer-reviewed study from Liu et al. provides the assessment of whether these design principles translated into robust and selective galectin-3 degradation in biologically relevant systems. These compounds are explicitly described in the patent as “degraders or inhibitors”, indicating an intended dual mode of action [7], whereas they are clearly referred to as heterobifunctional degraders in the very recent study [15]. However, their structural properties strongly support a targeted protein degradation design strategy and align closely with canonical PROTAC architectures. As such, these molecules represent one of the earliest examples of PROTAC-like approaches applied to lectin biology.

### 2.1. Galectin-Binding Moieties

**Figure 2:**
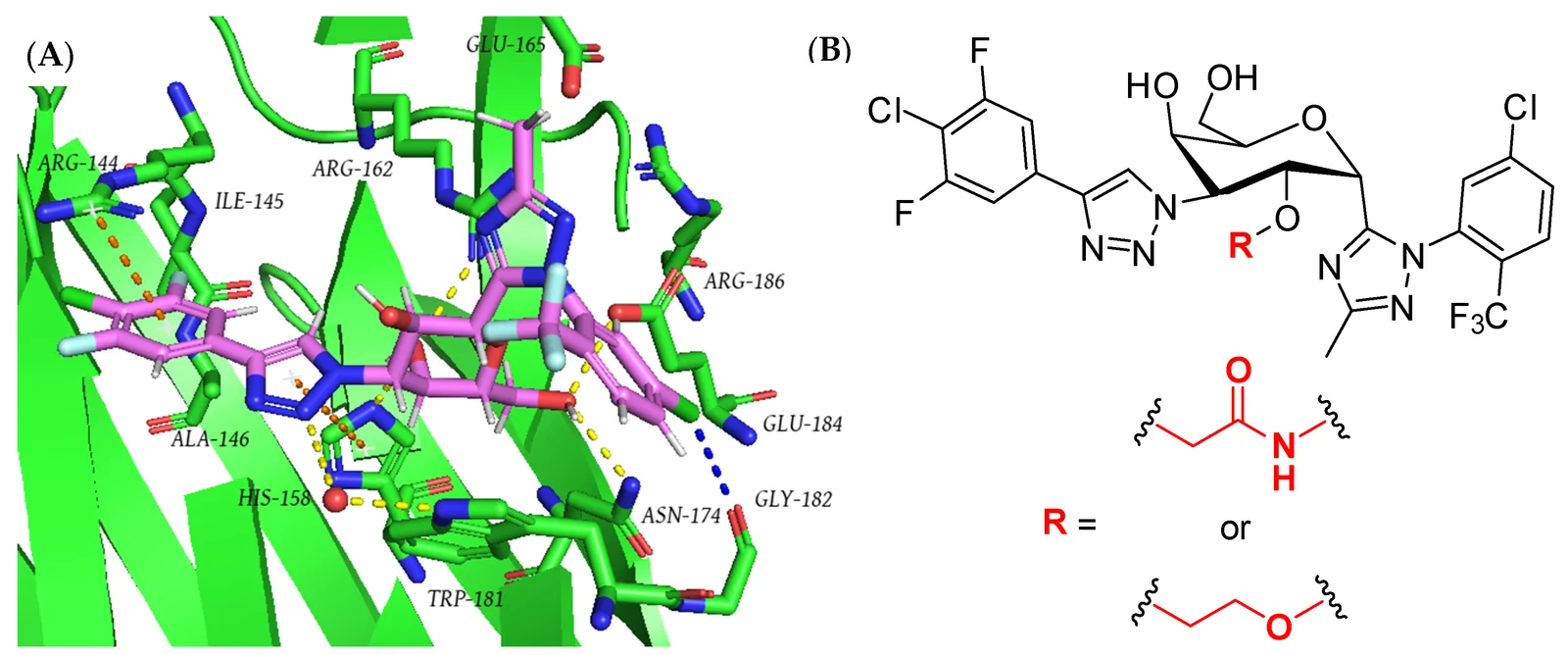
(**A**) A crystal structure of the human galectin-3 in complex with the galectin-3 inhibitor used in the synthesis of galectin-3-targeting PROTACs (PDB ID: 7XFA). Key interactions including a hydrogen bonding (depicted with yellow dashed lines) of the 4-OH with Arg162 and 6-OH with Asn174 and Glu184, cation-π interaction (depicted with orange dashed lines) between 4-chloro-3,5-difluorophenyl moiety at 3-OH and Arg144, π-π stacking (depicted with orange dashed lines) between the 1,2,3-triazolyl moiety and His158 and the interaction of chlorine of the anomeric 1,2,4-triazolylphenyl moiety and the carbonyl of Gly182 (depicted with blue dashed lines), and van der Waals contacts between the tetrahydropyran ring and Trp181 moiety are conserved. Position 2-OH points toward solvent and is therefore appropriate for linker attachment (PDB IDs: 7XFA, 5EXO, 5E89) [16,17,18]. (**B**) Galectin-3 binding part of bifunctional molecules presented in the patent. The most important interactions within the galectin-3 binding site are conserved.

The part of the ligand for POI is derived from advanced glycomimetic scaffolds optimised for high-affinity interaction with the galectin-3 CRD. This is crucial from a PROTAC design perspective, as it allows conjugation of a linker without affecting binding affinity in a negative context [13]. The structure of the ligand used in the synthesis of galectin-3-targeting PROTACs is presented in Figure 2.

### 2.2. E3 Ligase-Recruiting Ligand Choice

**Figure 3:**
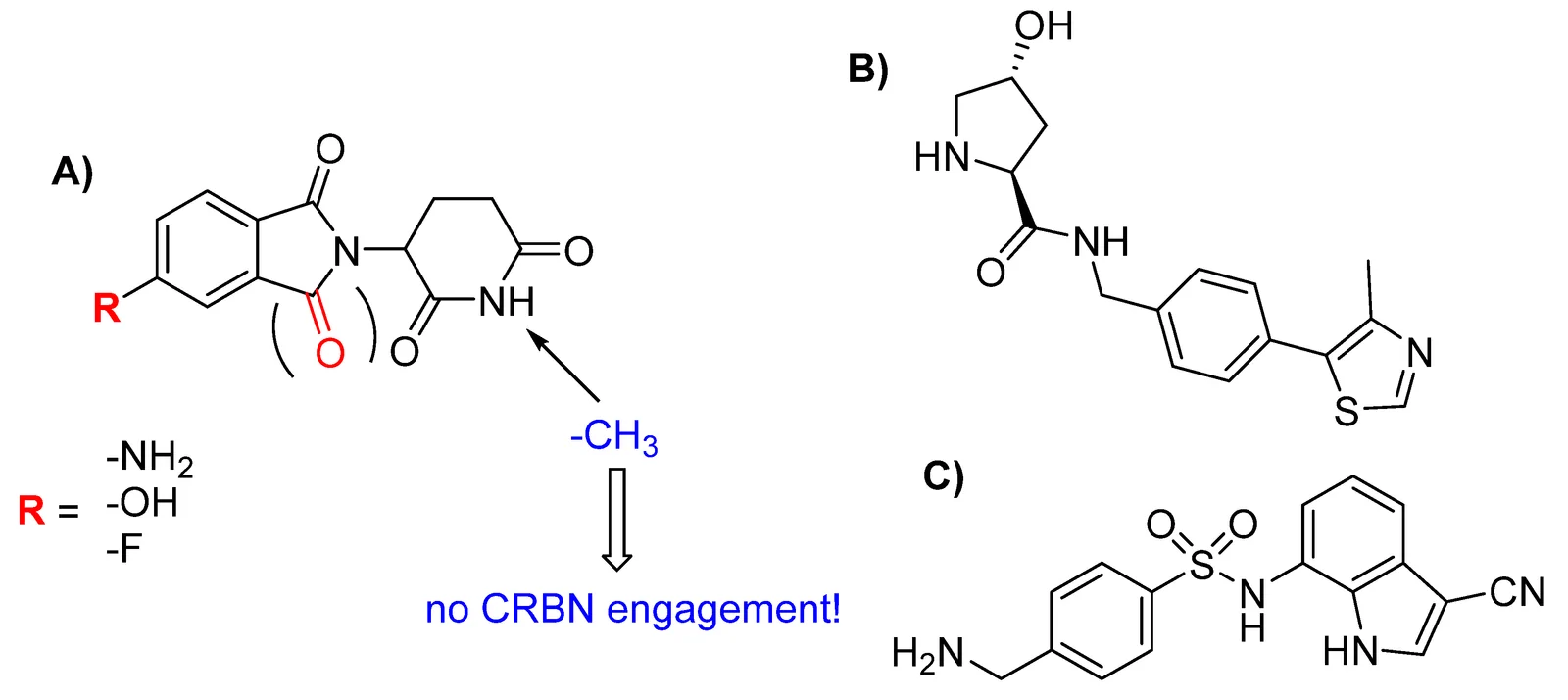
(**A**) E3 ligase-recruiting ligands used in the synthesis of galectin-3 bifunctional molecules, described in the patent. Methylated glutarimide of lenalidomide/pomalidomide is commonly used to block CRBN engagements and therefore serve as a negative control in degradation experiments. (**A**) lenalidomide/pomalidomide; (**B**) VHL ligand 1; (**C**) DCAF15 ligand (E7820-related).

The second very important component of the bifunctional molecules is the E3 ubiquitin ligase-recruiting moiety. The patent consistently incorporates glutarimide-containing scaffolds that are structurally analogous to immunomodulatory imide drugs (IMiDs), including isoindolinone and phthalimide-like motifs characteristic of CRBN binders such as thalidomide, lenalidomide, and pomalidomide derivatives (Figure 3), whereas in the peer-reviewed study only CRBN binders are described [15]. A couple of VHL (von Hippel–Lindau) and DCAF15-recruiting bifunctional molecules were also prepared, as disclosed in the patent [7]. However, CRBN and DCAF15 act as substrate receptors in CRL4 (cullin4A-RING) E3 ubiquitin ligase complexes rather than representing distinct E3 ligases.

The functional relevance of these structural elements is supported by CRBN target engagement assays, which demonstrate that the compounds retain the ability to bind CRBN in a cellular context (with the exception of VHL- and DCAF15-recruiting compounds). This is a critical hallmark of PROTAC functionality, as effective degradation depends on simultaneous engagement of both the POI and the recruited E3 ligase, followed by ternary complex formation. The exclusive focus on CRBN engagement is consistent with current trends in targeted protein degradation, where CRBN remains one of the most widely studied ligases due to its well-characterised small-molecule ligands and their favourable structural properties [19,20,21].

### 2.3. Linker Architecture and Design Considerations

**Figure 4:**
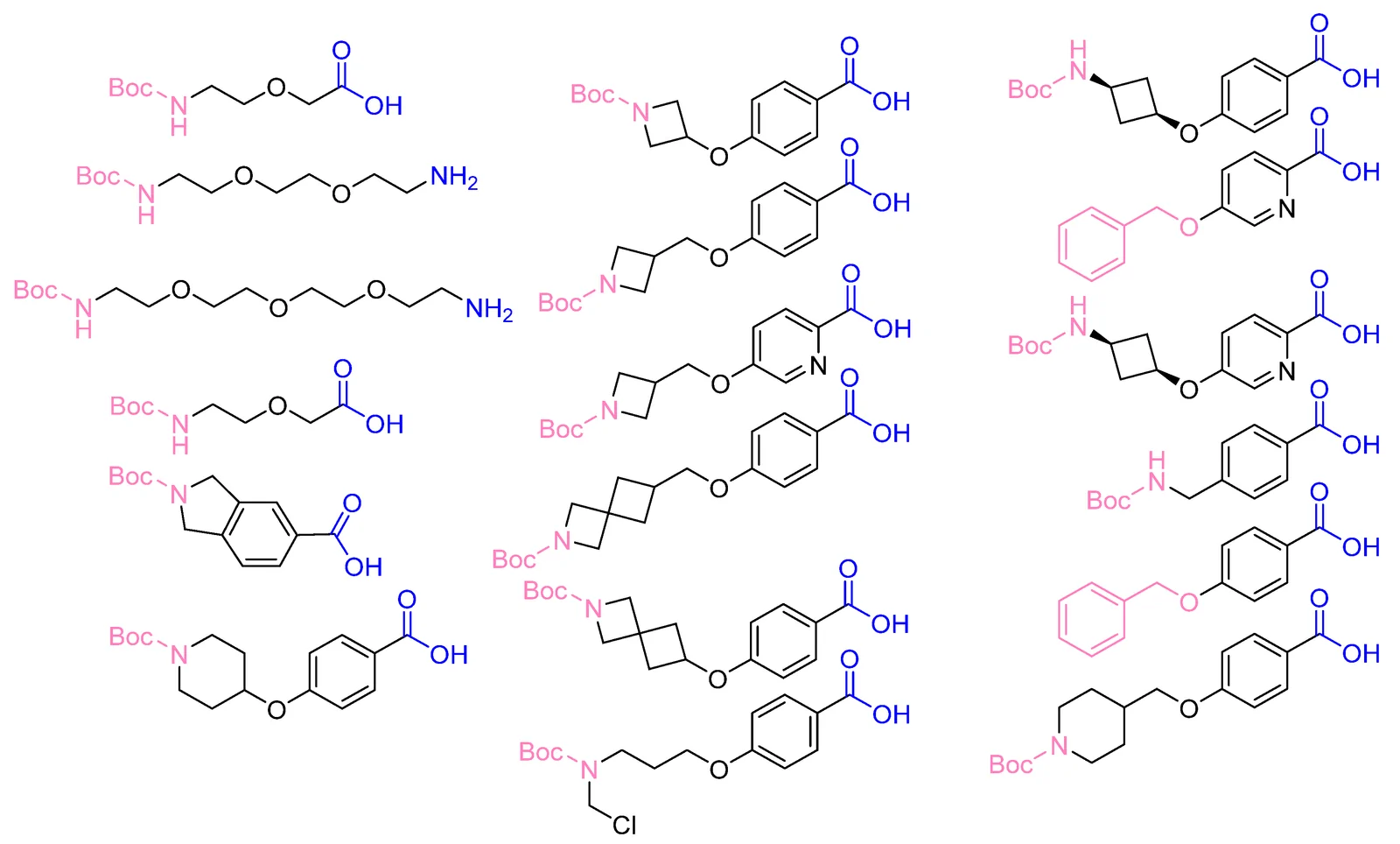
A collection of linkers used in the synthesis of galectin-3-targeting bifunctional molecules. Galectin-3 ligand (depicted in pink colour) and E3 ligase-recruiting ligand attachment points (depicted in blue colour) are shown.

A defining property of the disclosed compounds is the diversity of linker architecture connecting the ligand for the POI and the E3 ligase-recruiting ligand. The core linker design principles were already articulated in the patent, which describes a broad range of linkers, including alkylene chains, ethers, and amide-linked spacers, reflecting a systematic exploration of linker length, flexibility, and polarity (Figure 4). Linkers used in the patent often incorporate polyethylene glycol (PEG)-like motifs and polar functional groups that enhance flexibility and solubility. The subsequent peer-reviewed study did not fundamentally change these design principles; rather, it strengthened confidence in them by supporting their practical relevance. Such strategies are consistent with established PROTAC design principles, in which the linker plays a crucial role in modulating ternary complex formation, overall cellular activity, and pharmacokinetic properties as well [19,21]. Linker selection remains particularly challenging because of these requirements. Consequently, linker optimisation is frequently guided by a trial-and-error approach, as reflected in the wide structural diversity of linkers described in the patent. Linker design is a critical determinant of PROTAC activity. In particular, changes in linker rigidity and hydrophobicity can impact the cellular permeability, ternary complex formation, and subsequently degradation efficiency. This is especially relevant for galactose-based galectin-3 ligands, which typically possess a high polar surface area and therefore a reduced ability to pass through the cell membrane. In addition, an improperly chosen linker can lead to the “hook effect”, which is frequently observed in three-component systems. At PROTAC concentrations significantly above the DC_50_ value, autoinhibition of the formation of E3 ligase–PROTAC–target protein complexes occurs due to an increase in the amount of binary PROTAC–E3 ligase and PROTAC–target protein complexes, which subsequently leads to a decreased amount of ternary complex and reduced degradation of the target protein [22]. Moreover, the diverse linker choice strategy suggests significant effort was given to optimising the spatial orientation between the galectin-3 ligand moiety and the CRBN ligand, which is a key determinant of productive ubiquitination.

### 2.4. Functional Activity and Degradation Studies

**Figure 5:**
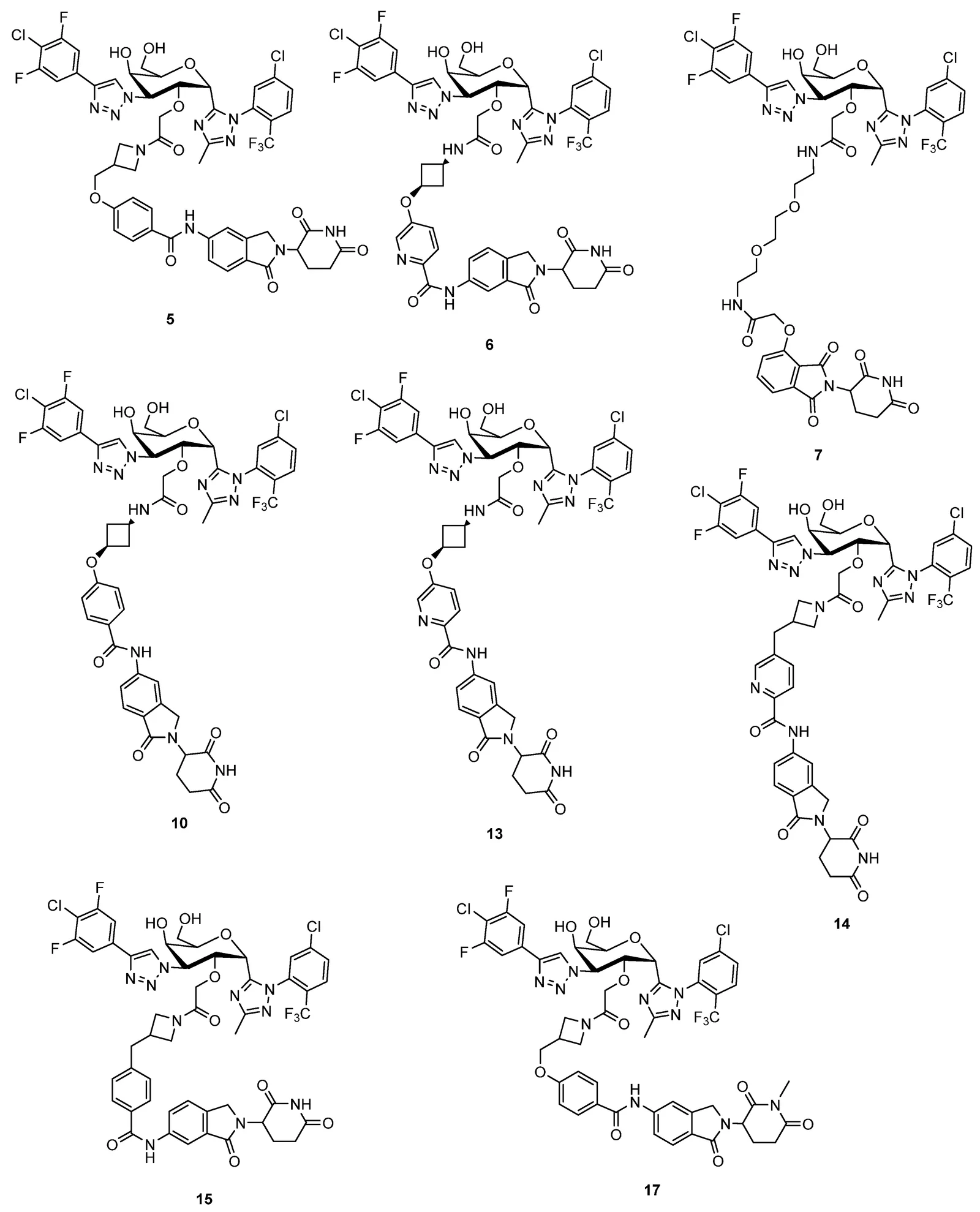
Structures of galectin-3-targeting PROTACs (**5**, **6**, **10**, **13**–**15**), non-degrading compound **7**, and negative control **17** [15].

The patent established the initial proof that galectin-3 could be targeted through a protein degradation strategy, whereas the recent peer-reviewed study provided the quantitative and proteome-wide validation required to evaluate the robustness of this approach. Importantly, the patent demonstrated that galectin-3 degradation was chemically and biologically plausible, while the peer-reviewed paper transformed that plausibility into a more convincing biological case for selective, potent, and efficient galectin-3 degradation. A CRBN BRET engagement assay and an hGal-3 HTRF assay were performed to determine the IC_50_ values for hCRBN and hGal-3 for each heterobifunctional molecule and are reported in both the patent and the peer-reviewed study. The reported galectin-3 and CRBN value correspond to assay-derived IC_50_ values rather than direct binding constants. Additionally, an enzyme-linked immunosorbent assay (ELISA) was used to quantify galectin-3 levels in THP-1 cells, a human monocyte cell line derived from leukaemia, suggesting an evaluation of targeted protein degradation rather than purely functional inhibition [7]. Disclosed in the patent, the observed reduction in galectin-3 levels measured by ELISA suggests there is a successful recruitment of the ubiquitin–proteasome system, although such data alone do not establish the underlying mechanism. Although the data is related to degradation efficacy, the patent does not explicitly report degradation parameters such as degradation potency (DC_50_) and maximal degradation (D_max_). Additionally, the patent does not explicitly demonstrate hallmarks of PROTAC-mediated degradation, such as direct evidence of target ubiquitination, proteasome dependence (carried out by proteasome inhibitors, such as MG132 and bortezomib/carfilzomib), or quantitative characterisation of ternary complex formation (evaluated by FRET-based or similar assays) [23,24]. Although the observed protein degradation is consistent with a degradation mechanism, alternative mechanisms, such as indirect modulation of protein expression or stability, cannot be fully excluded due to missing experiments. Therefore, several important questions remained unresolved after the patent disclosure, but the recent publication addressed some uncertainties. Although galectin-3 degradation has been demonstrated by Western blotting, representing one of the most important advances of the peer-reviewed study compared with the patent disclosure, studies of proteasome dependence and ternary complex formation are still needed to provide further mechanistic validation of the PROTAC approach. Besides that, experiments with neddylation inhibitors would be beneficial to prove that targeted degradation is strictly dependent on Cullin-RING E3 ligase, which is relevant for PROTACs that recruit CRBN or VHL. Nevertheless, the observed galectin-3 degradation is consistent with a PROTAC-mediated mechanism involving CRBN recruitment. In their biological studies, primary NHLF (Normal Human Lung Fibroblast) cells were treated with compounds in FGM-2 media (LONZA) for 72 h. Additionally, A549 (lung adenocarcinoma-derived cell line), LL29 (human lung fibroblasts derived from patient with idiopathic pulmonary fibrosis), and NIH/3T3 (mouse embryonic fibroblasts) cell lines were also used. Based on the potency, permeability, and cytotoxicity data, the effect on cellular galectin-3 levels of compounds **5**, **6**, **10**, and **13**–**15** (a direct comparison of selected compounds can be found in Table 1, while structures can be found in Figure 5) was monitored using Western blot, while *N*-methyl glutarimide **17** served as a negative control because the *N*-methyl moiety prevents the molecule from recruiting CRBN, thereby supporting evidence of CRBN recruitment. Furthermore, Liu et al. also presented galectin-3 degradation data in LL29 and A549 cells 72 h after treatment with compounds **5** and **10**, where both were effective in degrading galectin-3, with **5** being slightly less effective in the LL29 cell line (81% after 72 h). In particular, compound **5** demonstrated the most favourable overall activity profile and was subsequently studied in NHLF cells in a dose-dependent experiment, where key parameters of PROTAC activity were determined (DC_50_ = 4 nM, 72 h incubation) [15]. One of the most important advances is the proteome-wide selectivity analysis. Global proteomic analysis of compound **6** in THP-1 (acute monocyte leukaemia) cells demonstrated selective degradation of galectin-3 over more than 7800 quantified proteins, including other galectin family members, directly addressing one of the main risks in the design of lectin-targeted degraders. This study showed that **6** induced galectin-3 degradation in a dose-dependent manner after 48 h with a DC_50_ value of 10.7 nM, which is comparable to the data from Western blot analysis of compound **5**. The direct comparison of DC_50_ values between degraders **5** and **6** should be interpreted cautiously due to their different biological activity in various cell types under different conditions and readouts. Interestingly, comparison of active degraders with compounds **7** and **17** suggests that affinity for galectin-3 alone is insufficient to induce degradation, and that productive engagement of an E3 ligase and subsequent ternary complex formation is required.

## 3. Conclusions

Galectin-3-targeting degraders represent one of the most compelling recent examples of the expansion of targeted protein degradation beyond traditional intracellular target classes. Discovery of a successful degrader requires more than preservation of target affinity. The progression from patent disclosures to subsequent peer-reviewed validation demonstrates how rational optimisation of target binding, CRBN recruitment, linker architecture, and physicochemical properties can yield highly potent and selective degraders of a therapeutically relevant lectin. The identification of degraders achieving low nanomolar degradation potency highlights the extent to which productive ternary complex formation, rather than target binding alone, determines biological activity [25].

PROTAC-mediated targeting of galectin-3 could address several limitations of classical inhibitors. In principle, transient target engagement may be sufficient to trigger ubiquitination and proteasomal removal of the cytosolic proportion of the target protein. However, this approach depends on productive ternary complex formation, a requirement that is more demanding than binary binding [26,27]. PROTAC design also presents substantial challenges. Glycomimetic ligands and inhibitors are typically polar and structurally complex, potentially limiting cell permeability, metabolic stability, and conformational flexibility [8,28]. Importantly, cell permeability and metabolic stability were assessed by the authors using the parallel artificial membrane permeability assay (PAMPA, at pH = 5.5 and 7.4) and a liver microsomal stability assay, respectively. By introducing more hydrophobic and rigid linkers and lenalidomide as the CRBN recruiter, a series of cell-permeable and metabolically stable galectin-3 PROTACs were prepared. Permeability data were reported for only two compounds containing a PEG linker (compound **7** is given as an example in Figure 5) and were used, at least in part, to explain the lack of galectin-3 degradation observed for these compounds. Moreover, the relatively flat galectin-3 binding surface may not support cooperative ternary complex formation, potentially reducing ubiquitination efficiency and subsequent degradation [3,26]. Another limitation arises from the biology of galectin-3 itself. Galectin-3 is present in multiple cellular compartments and is also secreted into the extracellular space, where it regulates fibrosis and immune responses [1]. Because PROTAC activity is restricted to the intracellular ubiquitin–proteasome machinery, only a subset of galectin-3 pools is accessible to this mechanism, which raises an unresolved question as to whether selective depletion of intracellular galectin-3 is sufficient for achieving therapeutic efficacy, or whether the extracellular proportion of galectin-3 will limit the overall impact of this approach [5,6,12]. Based on the Western blot and global proteome analyses, Liu et al. identified highly potent and selective galectin-3 degraders, which offer a potentially attractive alternative to conventional inhibition. By removing galectin-3 itself, degraders may suppress both ligand-dependent and ligand-independent functions, providing a degree of pathway modulation that may be inaccessible through occupancy-based approaches. Whether this mechanistic advantage translates into superior therapeutic benefit remains an important question, but the concept is now supported by stronger experimental evidence than the data available in the patent. The relationship between intracellular galectin-3 degradation and extracellular galectin-3 biological functions remains incompletely understood. Although there is evidence that intracellular galectin-3 can be effectively depleted, it is still unclear whether this will reduce subsequent galectin-3 secretion and pre-existing extracellular galectin-3 and affect downstream signalling events regulated by extracellular galectin-3. To consider these compounds as therapeutic candidates, further studies will be required to determine in vivo efficacy, pharmacokinetic–pharmacodynamic relationships, and the long-term consequences of galectin-3 depletion in disease models. These studies will be essential for determining whether degradation offers meaningful advantages over traditional galectin-3 inhibition. Nevertheless, targeting intracellular galectin-3 via an event-driven mode of action remains an intriguing therapeutic opportunity, particularly in oncology, where the first-ever FDA-approved PROTAC has demonstrated clinical efficacy in patients with estrogen receptor-positive (ER+), HER2-negative (HER2-), *ESR1*-mutated advanced or metastatic breast cancer [29,30]. Further degraders may therefore provide a new strategy for modulating lectins whose intracellular and extracellular pools orchestrate distinct (patho)physiological functions. Galectin-1, -3, and -9 share high structural homology within their CRDs, making the development of highly selective galectin-3 ligands particularly challenging. Therefore, the selective degradation of galectin-3 reported by Liu et al., as demonstrated using quantitative proteomic analysis, represents an important milestone in the field of lectin-targeting PROTACs.

## Figures and Tables

**Table 1:** Direct comparison of binding affinity, metabolic stability and target depletion for compounds **5**, **6**, **7**, and **17**.

Compound	^a^ Galectin-3 Assay IC_50_ [nM]	^b^ CRBN Engagement Assay IC_50_ [nM]	^c^ Liver Microsomal Stability [%, h, r, m]	^d^ Target Depletion [%]
**5**	56 ± 8	399 ± 126	98/92/75	98
**6**	28, 55 ^e^	25 ± 12	100/96/93	94
**7**	20 ± 8	2850, 2085 ^e^	4.4/45/53	0
**17**	85 ± 7	>15,000	100/100/94	0

^a^ hGal-3 HTRF assay. ^b^ hCRBN BRET assay. ^c^ h–human liver microsomes, r–rat liver microsomes, m–mouse liver microsomes. ^d^ NHLF cells were incubated for 72 h with 1 μM concentration of compounds. ^e^ Mean values were determined from two experiments.

## Data Availability

No new data were created or analyzed in this study. Data sharing is not applicable to this article.

## Abbreviations

The following abbreviations are used in this manuscript:

BRET	Bioluminescence Resonance Energy Transfer
CRBN	Cereblon
CRD	Carbohydrate-Recognition Domain
CRL4	Cullin4A-Ring Ligase 4
ELISA	Enzyme-Linked Immunosorbent Assay
ESR1	Estrogen receptor 1
FDA	U. S. Food and Drug Administration
FRET	Förster Resonance Energy Transfer
HER2	Human Epidermal Growth Factor Receptor 2
HTRF	Homogenous Time-Resolved Fluorescence
NHLF	Normal Human Lung Fibroblast
PAMPA	Parallel Artificial Membrane Permeability Assay
POI	Protein of Interest
PROTACs	Proteolysis-Targeting Chimaeras
VHL	von Hippel–Lindau
